# Explaining Global Increases in Water Use Efficiency: Why Have We Overestimated Responses to Rising Atmospheric CO_2_ in Natural Forest Ecosystems?

**DOI:** 10.1371/journal.pone.0053089

**Published:** 2013-01-14

**Authors:** Lucas C. R. Silva, William R. Horwath

**Affiliations:** Biogeochemistry and Nutrient Cycling Laboratory, Department of Land, Air and Water Resources (LAWR), University of California Davis, Davis, California, United States of America; Centre National de la Recherche Scientifique, France

## Abstract

**Background:**

The analysis of tree-ring carbon isotope composition (δ^13^C) has been widely used to estimate spatio-temporal variations in intrinsic water use efficiency (iWUE) of tree species. Numerous studies have reported widespread increases in iWUE coinciding with rising atmospheric CO_2_ over the past century. While this could represent a coherent global response, the fact that increases of similar magnitude were observed across biomes with no apparent effect on tree growth raises the question of whether iWUE calculations reflect actual physiological responses to elevated CO_2_ levels.

**Methodology/Results:**

Here we use Monte Carlo simulations to test if an artifact of calculation could explain observed increases in iWUE. We show that highly significant positive relationships between iWUE and CO_2_ occur even when simulated data (randomized δ^13^C values spanning the observed range) are used in place of actual tree-ring δ^13^C measurements. From simulated data sets we calculated non-physiological changes in iWUE from 1900 to present and across a 4000 m altitudinal range. This generated results strikingly similar to those reported in recent studies encompassing 22 species from tropical, subtropical, temperate, boreal and mediterranean ecosystems. Only 6 of 49 surveyed case studies showed increases in iWUE significantly higher than predicted from random values.

**Conclusions/Significance:**

Our results reveal that increases in iWUE estimated from tree-ring δ^13^C occur independently of changes in ^13^C discrimination that characterize physiological responses to elevated CO_2_. Due to a correlation with CO_2_ concentration, which is used as an independent factor in the iWUE calculation, any tree-ring δ^13^C data set would inevitably generate increasing iWUE over time. Therefore, although consistent, previously reported trends in iWUE do not necessarily reflect a coherent global response to rising atmospheric CO_2_. We discuss the significance of these findings and suggest ways to distinguish real from artificial responses in future studies.

## Introduction

Anthropogenic activities have substantially altered atmospheric composition and climate with important implications for terrestrial biomes. Forest ecosystems are expected to be the most responsive, as tree species show higher increases in productivity and greater reductions in transpiration than any other functional type measured in large-scale (e.g. FACE sites) elevated CO_2_ experiments [1], [2]. These results are consistent with early chamber experiments and confirm that the intrinsic water use efficiency (iWUE) of trees, or the ratio between carbon uptake and water loss through transpiration, increases as stomatal conductance decreases in response to elevated CO_2_ [1]. In natural ecosystems, analyses of iWUE through the examination of carbon isotope ratios (δ^13^C) in tree-rings have also indicated increasing trends in response to rising atmospheric CO_2_ concentration [3]–[7]. However, contrasting with experimental studies, long-term changes in iWUE estimated from tree-ring δ^13^C have not been related to enhanced tree growth. Nearly identical increases in iWUE have been reported across biomes [8], but inconsistent tree growth responses and, in many cases, overall decline have been observed [9], [10].

These findings have generally been interpreted as evidence of large scale (warming-induced) drought stress, which despite CO_2_ stimulation, may lead to increased iWUE while limiting tree growth [6], [8]. Recent studies have, however, called attention to methodological issues that could hinder an accurate physiological interpretation of responses to rising CO_2_ based on the classic calculation of iWUE from tree-ring δ^13^C. For example, it has been suggested that due to a correlation with CO_2_, increases in iWUE would occur regardless of source to product (i.e. atmosphere to plant biomass) changes in isotopic fractionation that characterize physiological responses to environmental change [9]. If confirmed, this would indicate that temporal changes in iWUE estimated from tree-ring δ^13^C do not reflect actual shifts in either carbon uptake or water loss through transpiration. More importantly, it would imply that responses to rising CO_2_ have been globally overestimated, possibly explaining the lack of a clear effect on tree growth.

In this paper, we examine whether artifacts of calculation could explain increasing trends in iWUE reported in the recent literature. We use simulated (random) δ^13^C data and classic equations to determine how iWUE values relate to CO_2_ levels when there is no physiological change in source to product ^13^C fractionation. Based on simulated tree-ring δ^13^C data and actual atmospheric δ^13^C and CO_2_ measurements, we calculate changes in iWUE over the past century and across a wide altitudinal range. We then compare responses generated from simulated data with results from actual tree-ring δ^13^C obtained from the recent literature. We discuss our results and their implications for future research, focusing on the significance of previously observed responses and suggesting ways to validate changes in iWUE, testing the effect of atmospheric CO_2_ on natural forest ecosystems.

## Methods

### Water use efficiency calculation

The most widely used method to estimate changes in iWUE in natural ecosystems is the analysis of stable carbon isotope ratios (δ^13^C) in tree-rings [11]. The ^13^C/^12^C ratio in trees, and other C_3_ plants, is controlled at the leaf level by the ratio of intercellular (C_i_) to ambient (C_a_) CO_2_ concentrations. If C_i_ is high relative to C_a_, strong discrimination against ^13^C yields isotopically light (^12^C enriched) biomass. Conversely, if C_i_ is low there is less discrimination against ^13^C resulting in higher δ^13^C values. Regardless of growth rates or net changes in productivity, any change in carboxylation and/or stomatal conductance that alters C_i_/C_a_ is recorded as a change in δ^13^C [12], which in the case of tree species that produce annual growth rings can be used to reconstruct physiological changes over long periods of time [11], [13]. Because atmospheric δ^13^C also varies over time, tree-rings must be analyzed in relation to atmospheric ^13^C abundance at the moment of its assimilation. For example, anthropogenic CO_2_ emissions have decreased the δ^13^C composition of the atmosphere [14], as fossil fuels (depleted in ^13^C) result in a reduction of ^13^CO_2_ relative to ^12^CO_2_. Therefore, physiological changes that occur in coincidence with anthropogenic emissions can only be assessed after changes in atmosphere to plant biomass discrimination are accounted for, which is done as follows [15]:
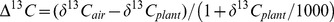
(1)where Δ^13^C is discrimination against ^13^C, δ^13^C_air_ is the carbon isotope ratio of air (the source) and δ^13^C_plant_ is the carbon isotope ratio of the product (plant biomass). To be translated into physiologically relevant information Δ can be expressed as:
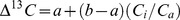
(2)where *a* is the discrimination against ^13^CO_2_ during diffusion through the stomata (−4.4‰) and *b* is the net discrimination due to carboxylation (−27‰). Real increases in carboxylation rates or reduction in conductance, expected in responses to rising CO_2_ levels, would result in a distinct shift in Δ^13^C [12]. Following Fick's first law (A = *g*CO_2_(C_a_−C_i_)), Δ^13^C values can be converted into plant's intrinsic water use efficiency (iWUE) at the moment of biomass production [11] as follows:
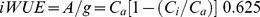
(3)where A is net carboxylation, *g* is the leaf stomatal conductance and 0.625 is the relation between conductance for CO_2_ molecules and water vapor.

### Calculating iWUE from simulated δ^13^C

To test whether correlations involved in the calculation of iWUE could explain systematic (non-physiological) increases in response to rising CO_2_, we used Monte Carlo randomizations [16], generating one thousand synthetic δ^13^C_plant_ data sets that are similar (vary within the same range) to observed tree-ring data. This method allowed us to build a probability distribution and study what features of the distribution are essential for describing previously reported (observed) patterns. The underlying assumption is that the simulated distribution represents the observed data well enough so that variation in randomized and measured data is the same. Real tree-ring δ^13^C ranges from about −20 to −30‰, which typically corresponds to water-stressed and unstressed conditions respectively. To represent different portions of this spectrum, we used simulations of δ^13^C ranging from −20 to −21‰, −25 to −26‰ and −29 to −30‰. At each range we calculated ^13^C discrimination (Δ) in relation to a constant atmospheric δ^13^C composition (−8‰), determining changes in iWUE in response to atmospheric CO_2_ as the only varying factor.

### Changes over time and with altitude

To translate the outcomes of our simulated data into trends comparable with those reported in the recent literature, we calculated changes in iWUE from 1900 to present and across a 4000 m altitudinal range. To calculate iWUE over time we used actual atmospheric values of δ^13^C_air_ and C_a_ [14], [15] and simulated tree-ring δ^13^C data sets. To describe changes in CO_2_ partial pressure and ^13^C content with altitude, we relied on well-established relationships between δ^13^C_plant_ and C_a_ partial pressure. Globally, δ^13^C_plant_ increases ∼0.8‰ for every 1000 m of altitudinal gain, a pattern that holds independently of plant species [17], [18]. We used average iWUE obtained from simulated δ^13^C data coupled with ^13^C enrichment expected with altitude (δ^13^C_Alt_ = 0.0008*Alt+δ^13^C_plant_). We then constructed a simple three-dimensional model of changes in iWUE over the past century and across altitudinal gradients as follows:
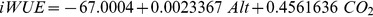
(4)where temporal changes in iWUE (µmol mol^−1^) are a function of both CO_2_ (ppm) and altitude (m). We then regressed annual percent increases in iWUE calculated from simulated data sets (predicted) against iWUE measured from tree-ring δ^13^C (observed) in 49 recent studies encompassing 22 species from tropical, subtropical, temperate, boreal and mediterranean ecosystems (Table S1). Significant differences were tested using the difference between observed iWUE distributions of each surveyed case study in relation to predicted iWUE, using 95% confidence levels. Statistical analysis and Monte Carlo simulations were performed using JMP software for Macintosh, version 10.

## Results and Discussion

### iWUE calculated from simulated data

**Figure 1:**
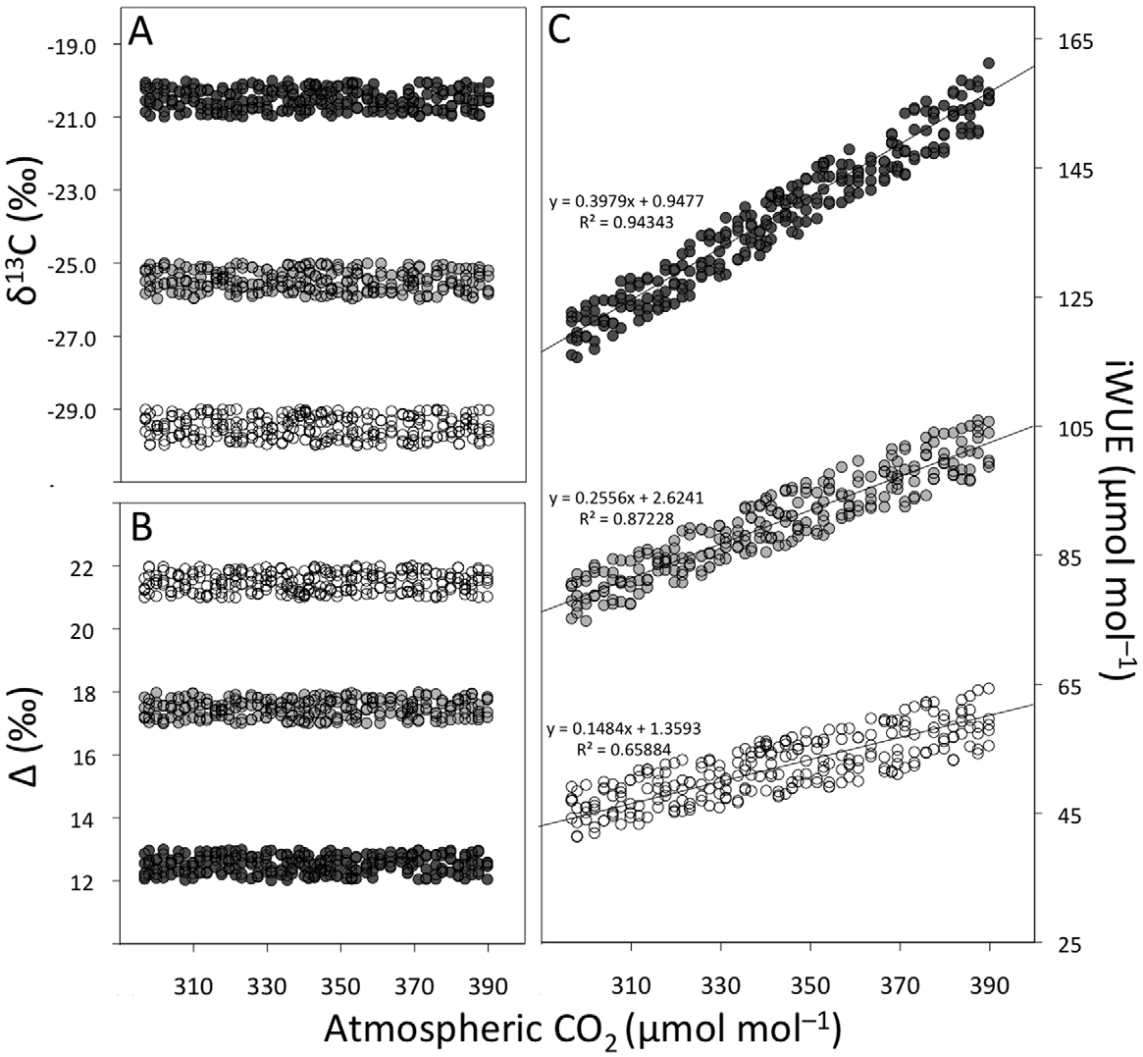
Simulated δ^13^C data, randomized within three different ranges: −20 to −21‰ (dark grey circles); −25 to −26‰ (light grey circles) and −29 to −30‰ (white circles). (A). Source to product ^13^C discrimination calculated from simulated δ^13^C according to eq. 2 using atmospheric δ^13^C value of −8‰ (B). Estimated iWUE calculated from simulated δ^13^C and Δ following eq. 3 (C). Note that significant (P<0.001) positive relationships between iWUE and atmospheric CO_2_ occur despite no changes in δ^13^C and Δ.

**Figure 2:**
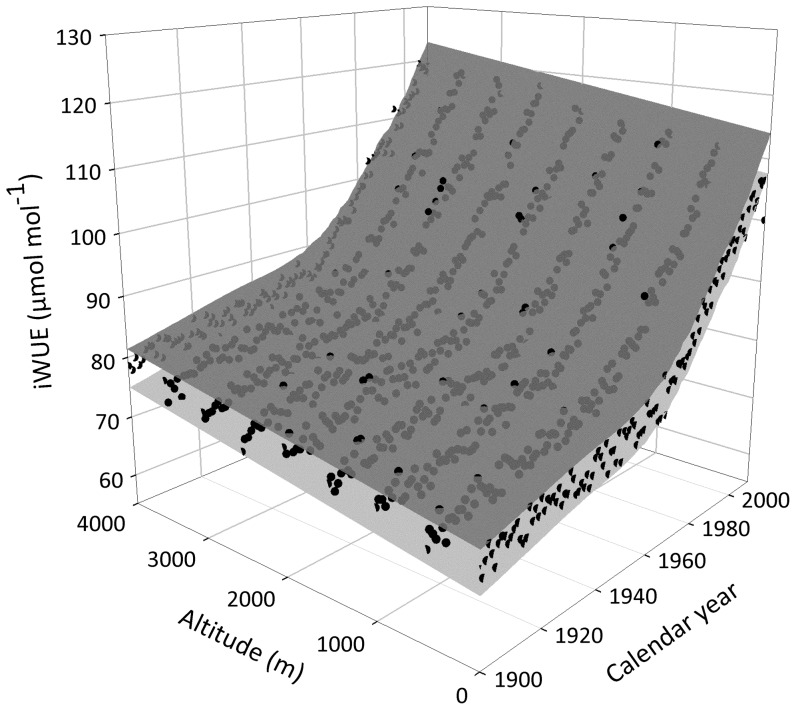
Average increase in intrinsic water use efficiency (iWUE) calculated from simulated δ^13^C data sets (**Fig. 1**) using classic equations (**eqs. 1** to **3**) and actual values of atmospheric δ^13^C and CO_2_ concentrations [**14**], [**15**] **.** Black circles represent predicted iWUE values and surfaces show upper and lower (95%) confidence intervals for changes in iWUE estimated through time (calendar year) and across a 4000 m altitudinal gradient as function of CO_2_ according to eq. 4.

Simulated δ^13^C data (Fig. 1A), used in place of actual tree-ring δ^13^C, generated highly significant positive relationships between iWUE and atmospheric CO_2_. Despite constant atmosphere to biomass discrimination (Δ) (Fig. 1B), which is a universal measure of physiological responses to environmental change [19], iWUE calculated from simulated δ^13^C always increased along with CO_2_ levels (Fig. 1C). Randomizations within constant δ^13^C ranges, such as the ones used here, reflect what would be observed if C_i_ increased proportionally with C_a_ or, in other words, if C_i_/C_a_ was kept constant as C_a_ rises. This corresponds to the most conservative theoretical scenario for increases in iWUE [20], where δ^13^C and Δ do not change (eqs. 1 and 2) and changes in iWUE are derived from variation in CO_2_ alone (eq. 3). The graphic (average) expression of simulated δ^13^C as estimated iWUE shows increases of more than 40 µmol mol^−1^ over the last century and about 20 µmol mol^−1^ across altitudinal gradients (Fig. 2). This demonstrates that large increases in iWUE would be observed in association with calendar year and altitude in any data set, regardless of actual physiological responses. Plant regulation of A and/or *g*, which could actively keep C_i_ constant as CO_2_ levels rise leading to increased iWUE and δ^13^C due to reduced C_i_/C_a_ and Δ [3], [7], is not represented by our δ^13^C randomizations. If such regulations were to be considered in a theoretical scenario of constant C_i_, increases in iWUE greater than those generated by our model would be observed [20].

### Observed versus predicted responses

**Figure 3:**
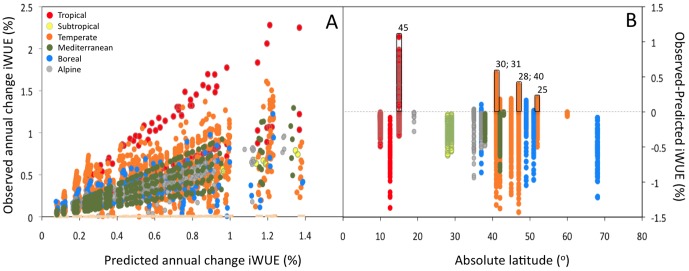
Relationships between iWUE predicted based on simulated δ^13^C values (**Fig. 1**), corrected for changes with calendar year and altitude (**Fig. 2**), and iWUE observed from in 49 case studies. (A). Difference between observed and predicted change in iWUE (B). Note that only six case studies, namely 25, 28, 30, 31, 40 and 45 (Table S1) showed increases in iWUE significantly higher than predicted from simulated δ^13^C data (upper 95% confidence interval across latitudes in panel B = 0.3%).

Predicted responses based on simulated data sets generate results remarkably similar to those determined from actual tree-ring δ^13^C data, suggesting that previously reported trends in iWUE do not represent an implicit physiological response to rising CO_2_. Several case studies [3]–[7] (see also supplementary material) and meta-analyses of tree-ring δ^13^C [8], [9] have concluded that synchronous increases in iWUE, typically 0.3–0.5% per year, have occurred over the past century. Annual changes in iWUE calculated from simulated δ^13^C show increases of similar magnitude (Fig. 2), which are linearly related with empirical data (Fig. 3A). Global analyses of tree-rings have identified distinct responses associated with latitude [9]. However, when simulated results are subtracted from observed iWUE data, only 6 out of 49 case studies show significant increases; furthermore, all latitudinal trends disappear (Fig. 3B). These findings suggest that an overall acclimation, rather than enhanced efficiency, was the predominant response over the past century across biomes.

Mechanisms controlling leaf [21], [22] and ecosystem [1], [23] level acclimation have been described experimentally and both productivity and water use responses have been shown to vary with length of exposure to elevated CO_2_. Reduced/acclimated stimulation of net carbon assimilation (A) has been generally attributed to decreased carboxylation velocity and investment in Rubisco [1]. Similarly, long-term hydraulic acclimation under elevated CO_2_ allows plants to reduce stomatal conductance (*g*) less than plants growing under ambient CO_2_ [24]. If under natural conditions both A and *g* acclimate as suggested by experimental results, maintenance rather than continuous increases in iWUE should be observed as a result of decadal to centennial CO_2_ stimulation. Our comparisons between predicted and observed iWUE data suggest that this occurred in most case studies surveyed here. The few cases that showed increases in iWUE significantly higher than predicted by simulated data include deciduous and coniferous species, growing in temperate and tropical regions ranging from 100 to about 1600 m asl (Table S1). No obvious reason could be found to explain why these studies showed higher iWUE than predicted. Nevertheless, the observed idiosyncratic trends suggest the importance of site- and species-specific responses.

### Significance and implications for future research

To date, divergent patterns found between iWUE and growth have been interpreted as evidence of warming-induced water stress, which could explain both reduced productivity and enhanced water efficiency [3]–[8]. Where growth rates appear to be positively related to iWUE, results have been interpreted as evidence of CO_2_ stimulation [6], [9]. However, here we show that due to an artifact of calculation systematic increases in iWUE would be inevitably generated by any δ^13^C data set and, as such, are not causally linked to either growth decline or stimulation. On the basis of isotope theory, δ^13^C in tree-rings varies in response to changes in conditions that affect processes controlling photosynthesis and/or transpiration during the year in which the ring was formed [11]. Hence, analysis of δ^13^C in tree-rings offers valuable information to study how environmental changes affect tree development and water use over time. While we agree with the theory and the well-established association found between δ^13^C and leaf-level physiological processes [12], [17], [18], our results show that the extrapolation of this association from tree-ring δ^13^C should be reevaluated.

Stable isotopes in tree-rings have now been measured in many parts of the world. Though substantial, inter- and intra-specific differences in ^13^C discrimination [3], [5], [7], [13] and variation across altitudinal and latitudinal gradients [9], [17], [18] have been overlooked in most iWUE studies. Global estimates of iWUE integrated over the past decades, without accounting for such variability, suggest that increases of the same magnitude with no significant differences occurred across biomes [8]. Our simulations, however, show that these consistent trends in iWUE cannot be interpreted as a coherent global response to rising CO_2_. Most of the responses observed in the surveyed studies could be explained by a correlation with CO_2_ (Fig. 3), suggesting that physiological responses have been overestimated.

Complementary methods should be used in combination with iWUE analysis to distinguish real from artificial effects and improve spatio-temporal scaling of the impacts of climate and atmospheric change on terrestrial systems. The analysis of source to product ^13^C fractionation combined with tree radial growth, or the calculation of response contrast based on cumulative changes in iWUE and productivity, can be used to distinguish between CO_2_ fertilization effects and warming-induced stress [9]. Other isotopic tracers related to water use, such as δ^18^O, could also be used for this purpose [11]. While δ^13^C does not provide any indication of whether changes in iWUE are due to changes in photosynthesis or transpiration, if tree-ring δ^18^O increases with δ^13^C this would indicate that changes in iWUE were caused by reductions in stomatal conductance rather than increases in photosynthesis [5], [25]. If source to product ^13^C discrimination, growth or δ^18^O data are not available, comparisons between empirically determined iWUE and theoretical baselines generated from simulated data sets (Fig. 2, but see also [20]), could be used to control for artificial trends in future studies.

## Supporting Information

Table S1 **List of case studies that reported physiological changes in response to rising atmospheric CO_2_.** (*) Indicates studies where annual percent change in iWUE was reported in the original text; (**) indicates studies where only δ^13^C series were presented. In all case studies iWUE was determined based on tree-ring δ^13^C following classic calculations (eq. 1 to 3) and using real values of atmospheric δ^13^C and CO_2_ concentrations [14], [15]. The equation that best describes the relationship between iWUE and CO_2_ series in each case study and the period of the observation are shown. From these relationships annual percent changes was calculated to project iWUE values over the past century (Fig. 3).
(DOC)
