# Supplementary material for: Explaining Global Increases in Water Use Efficiency: Why Have We Overestimated Responses to Rising Atmospheric CO2 in Natural Forest Ecosystems?
Source: PLoS One. 2013 Jan 14;8(1):e53089. doi: 10.1371/journal.pone.0053089 (PMC3544798; doi:10.1371/journal.pone.0053089)
Supplement: Table S1 — List of case studies that reported physiological changes in response to rising atmospheric CO2. (*) Indicates studies where annual percent change in iWUE was reported in the original text; (**) indicates studies where only δ13C series were presented. In all case studies iWUE was determined based on tree-ring δ13C following classic calculations (eq. 1 to 3) and using real values of atmospheric δ13C and CO2 concentrations [14], [15]. The equation that best describes the relationship between iWUE and CO2 series in each case study and the period of the observation are shown. From these relationships annual percent changes was calculated to project iWUE values over the past century (Fig. 3). (DOC) [file pone.0053089.s001.doc]

**Table S1**

**Table S1**

**References**

Nock, C. a et al. Long-term increases in intrinsic water-use efficiency do not lead to increased stem growth in a tropical monsoon forest in western Thailand. Global Change Biology 17, 1049-1063 (2011).

Silva, L.C.R., Anand, M. & Leithead, M.D. Recent Widespread Tree Growth Decline Despite Increasing Atmospheric CO2. PLoS ONE 5, 7 (2010).

Andreu-Hayles, L. et al. Long tree-ring chronologies reveal 20th century increases in water-use efficiency but no enhancement of tree growth at five Iberian pine forests. Global Change Biology 17, 2095-2112 (2011).

Peñuelas, J., Hunt, J.M., Ogaya, R. & Jump, A.S. Twentieth century changes of tree-ring δ 13 C at the southern range-edge of Fagus sylvatica: increasing water-use efficiency does not avoid the growth decline induced by warming at low altitudes. Global Change Biology 14, 1076-1088 (2008).

Peñuelas, J., Canadell, J.G. & Ogaya, R. Increased water-use efficiency during the 20th century did not translate into enhanced tree growth. Global Ecology and Biogeography 20, 597-608 (2010).

Waterhouse, J.S. et al. Northern European trees show a progressively diminishing response to increasing atmospheric carbon dioxide concentrations. Quaternary Science Reviews 23, 803-810 (2004).

Bert, D., Leavitt, S.W. & Dupouey, J.-luc Variations of Wood δ13C and Water-Use Efficiency of Abies Alba During the Last Century. America 78, 1588-1596 (2011).

Holzkämper, S., Kuhry, P., Kultti, S., Gunnarson, B. & Sonninen, E. Stable Isotopes in Tree Rings as Proxies for Winter Precipitation Changes in the Russian Arctic over the Past 150 Years. Geochronometria 32, 37-46 (2008).

Liu, X. et al. Response and dendroclimatic implications of δ 13 C in tree rings to increasing drought on the northeastern Tibetan Plateau. Journal of Geophysical Research 113, 1-11 (2008).

Liu, X. et al. Species-dependent responses of juniper and spruce to increasing CO2 concentration and to climate in semi-arid and arid areas of northwestern China. Plant Ecology 193, 195-209 (2007).

Liu, X. Temperature variations recovered from tree-rings in the middle Qilian Mountain over the last millennium. Science in China Series D 48, 521 (2005).

Shao, X. Reconstruction of precipitation variation from tree rings in recent 1000 years in Delingha, Qinghai. Science in China Series D 48, 939 (2005).

Liang, E. et al. The 1920S Drought Recorded by Tree Rings and Historical Documents in the Semi-Arid and Arid Areas of Northern China. Climatic Change 79, 403-432 (2006).

Hietz, P., Wanek, W. & Dünisch, O. Long-term trends in cellulose delta13 C and water-use efficiency of tropical Cedrela and Swietenia from Brazil. Tree physiology 25, 745-52 (2005).

Duquesnay, a, Breda, N., Stievenard, M. & Dupouey, J.L. Changes of tree-ring delta13C and water-use efficiency of beech (Fagus sylvatica L.) in north-eastern France during the past century. Plant, Cell and Environment 21, 565-572 (1998).

Ballantyne, a P., Baker, P. a, Chambers, J.Q., Villalba, R. & Argollo, J. Regional Differences in South American Monsoon Precipitation Inferred from the Growth and Isotopic Composition of Tropical Trees*. Earth Interactions 15, 1-35 (2011).

Reyes M.A.B. Isotopos estables de 18O, 2H y 13C en anillos de crescimiento de coniferas en el eje neovolcanico transversal de Mexico. M.Sc. Dissertation. Montecillo Forest Institute (2011)

L Tang K., Feng X., and Funkhouser G. S. The δ13C of tree rings in full-bark and strip-bark bristlecone pine trees in the White Mountains of California. Global Change Biology 5(1), 33–40 (1999).
